# Development of a pharmacorphore model for pharmacological chaperones targeting mutant trafficking-deficient CNG channels

**DOI:** 10.1186/1758-2946-5-S1-O18

**Published:** 2013-03-22

**Authors:** Charlotta PI Schärfe, Joachim Taeger, Peggy Reuter, Nina M Fischer, Jens Krüger, Bernd Wissinger, Oliver Kohlbacher

**Affiliations:** 1 Applied Bioinformatics, Center for Bioinformatics, Quantitative Biology Center and Department of Computer Science, University of Tübingen, Tübingen, 72076, Germany; 2 Molecular Genetics Laboratory, Institute for Ophthalmic Research, Center for Ophthalmology, Tübingen, 72076, Germany

## 

Complete colorblindness (achromatopsia) is caused by autosomal recessively inherited mutations in the retinal phototransduction pathway, predominantly in the CNGA3- and CNGB3-subunit of the cyclic nucleotide-gated (CNG) channels in cone photoreceptors. CNGA3, which is mutated in about 25% of the achromatopsia patients, mainly harbors missense mutations which frequently impair the folding and/or trafficking of the mutant CNGA3-channels [1].

Pharmacological chaperones stabilizing the folding of the mutant protein may be used to overcome this folding-/trafficking-deficiency. More than 50 compounds were evaluated in their ability to restore signal transduction using a calcium imaging-based bioassay utilizing the CNGA3-mutant E228K [2]. With this data we created several pharmacophore models using Schrödinger Phase [3], which describe the chemical features of potential pharmacological chaperones targeting achromatopsia.

**Figure 1:**
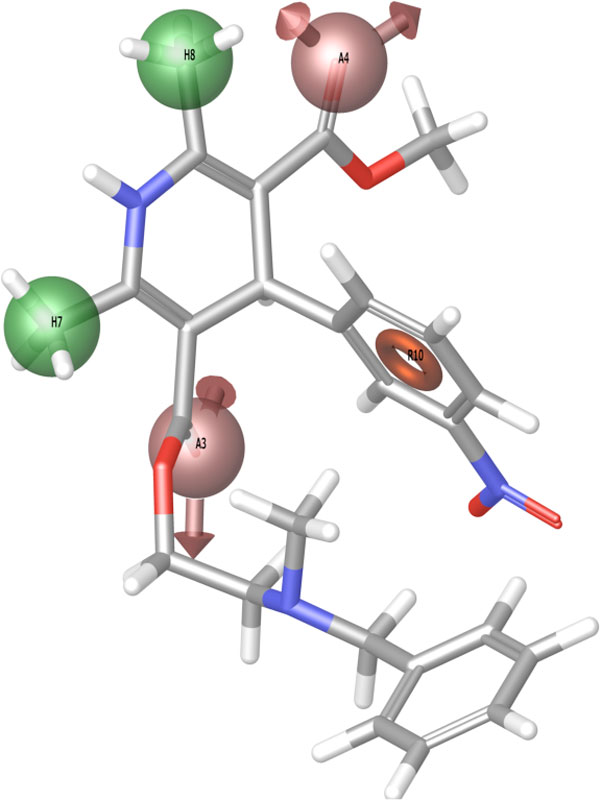
**Pharmacophore depicting potential features of CNG channel-chaperones**.

We used several approaches leading to different pharmacophore hypotheses:

a) Training with the complete set of experimental data (see Figure 1)

b) Training with only dihydropyridines since this group showed the highest experimental activity, and

c) Training with a data set excluding dihydropyridines.

Our in-house database TueScreen, which includes ZINC12 [4], was screened to identify potentially active compounds. As a result, several potential molecule classes could be found that may be useful as pharmacological chaperones to improve folding/trafficking of mutant CNG-channels. We will experimentally validate these predictions in a calcium imaging-based bioassay.
